# Protocol for assessing maternal, environmental and epigenetic risk factors for dental caries in children

**DOI:** 10.1186/s12903-015-0143-2

**Published:** 2015-12-29

**Authors:** Surani Fernando, David J. Speicher, Mahmoud M. Bakr, Miles C. Benton, Rodney A. Lea, Paul A. Scuffham, Gabor Mihala, Newell W. Johnson

**Affiliations:** School of Dentistry and Oral Health, Gold Coast Campus, Griffith University, Gold Coast, 4222 Queensland Australia; Population and Social Health Research Program, Menzies Health Institute Queensland, Gold Coast Campus, Griffith University, Gold Coast, 4222 Queensland Australia; Molecular Basis of Disease Program, Menzies Health Institute Queensland, Griffith Health Institute, Gold Coast Campus, Griffith University, Gold Coast, 4222 Queensland Australia; Institute of Health and Biomedical Innovation, Queensland University of Technology, Kelvin Grove, GPO Box 2434, Brisbane, 4001 Queensland Australia

**Keywords:** Dental caries, Children, Risk, Environment, Genetic, Epigenetic

## Abstract

**Background:**

Expenditure on dental and oral health services in Australia is $3.4 billion AUD annually. This is the sixth highest health cost and accounts for 7 % of total national health expenditure. Approximately 49 % of Australian children aged 6 years have caries experience in their deciduous teeth and this is rising. The aetiology of dental caries involves a complex interplay of individual, behavioural, social, economic, political and environmental conditions, and there is increasing interest in genetic predisposition and epigenetic modification.

**Methods:**

The Oral Health Sub-study; a cross sectional study of a birth cohort began in November 2012 by examining mothers and their children who were six years old by the time of initiation of the study, which is ongoing. Data from detailed questionnaires of families from birth onwards and data on mothers’ knowledge, attitudes and practices towards oral health collected at the time of clinical examination are used. Subjects’ height, weight and mid-waist circumference are taken and Body Mass Index (BMI) computed, using an electronic Bio-Impedance balance. Dental caries experience is scored using the International Caries Detection and Assessment System (ICDAS). Saliva is collected for physiological measures. Salivary Deoxyribose Nucleic Acid (DNA) is extracted for genetic studies including epigenetics using the SeqCap Epi Enrichment Kit. Targets of interest are being confirmed by pyrosequencing to identify potential epigenetic markers of caries risk.

**Discussion:**

This study will examine a wide range of potential determinants for childhood dental caries and evaluate inter-relationships amongst them. The findings will provide an evidence base to plan and implement improved preventive strategies.

## Background

Dental caries affects children and adults alike all over the world. It is a disease of the poor as well as of the rich. Largely preventable, it remains the most prevalent chronic disease among children with a significant impact on individuals, families and society. It has recently been reported that 2.4 billion people globally have untreated dental caries [1]. More than 40 % of preschool and primary school children in Western industrialised countries and other middle income countries, including children in the United States of America [2], Sweden [3], Brazil [4] and Australia [5] experience a high prevalence of dental caries. Despite progress made in caries control over the years by the protective effects of fluoride, increased efforts in oral health promotion, widespread health education, and remarkable advances in treatment options, dental caries remains the most common chronic childhood disease [1]. Among Australian children aged 6–7 years the prevalence of dental caries (treated and untreated combined) was 32.4 % in 2011 [6]. This figure varies by state from 26.5 % in New South Wales to 43.8 %, in Queensland (Table 1) [6]. According to the Queensland Child Oral Health Survey in 2012, 49.5 % of children aged 5–10 had experienced dental caries [7]. More alarmingly, among South East Queensland children this figure was just over 50 % [7]. The mean decayed missing and filled teeth index (dmft) for the same child population was 2.0 with 0.8 teeth being untreated [7]. This worsening situation is despite substantial resources being allocated for prevention and treatment of oral diseases [8].

**Table 1:** Percentage and 95 % Confidence Interval of 6–7 year old children with caries experience in Australia in 2011

State	Caries experience
Australian Capital Territory	32.4 (21.8, 43.1)
South Australia	29.8 (24.6, 35.0)
Western Australia	35.5 (31.1, 40.0)
Victoria	30.0 (27.3, 32.7)
New South Wales	26.5 (28.2, 46.3)
Tasmania	37.3 (40.6, 47.1)
Queensland	43.8 (40.6, 47.1)
Northern Territory	37.8 (15.7, 42.1)
All States	32.4 (31.0, 33.8)

Source Lucas et al. [6]

**Fig. 1:**
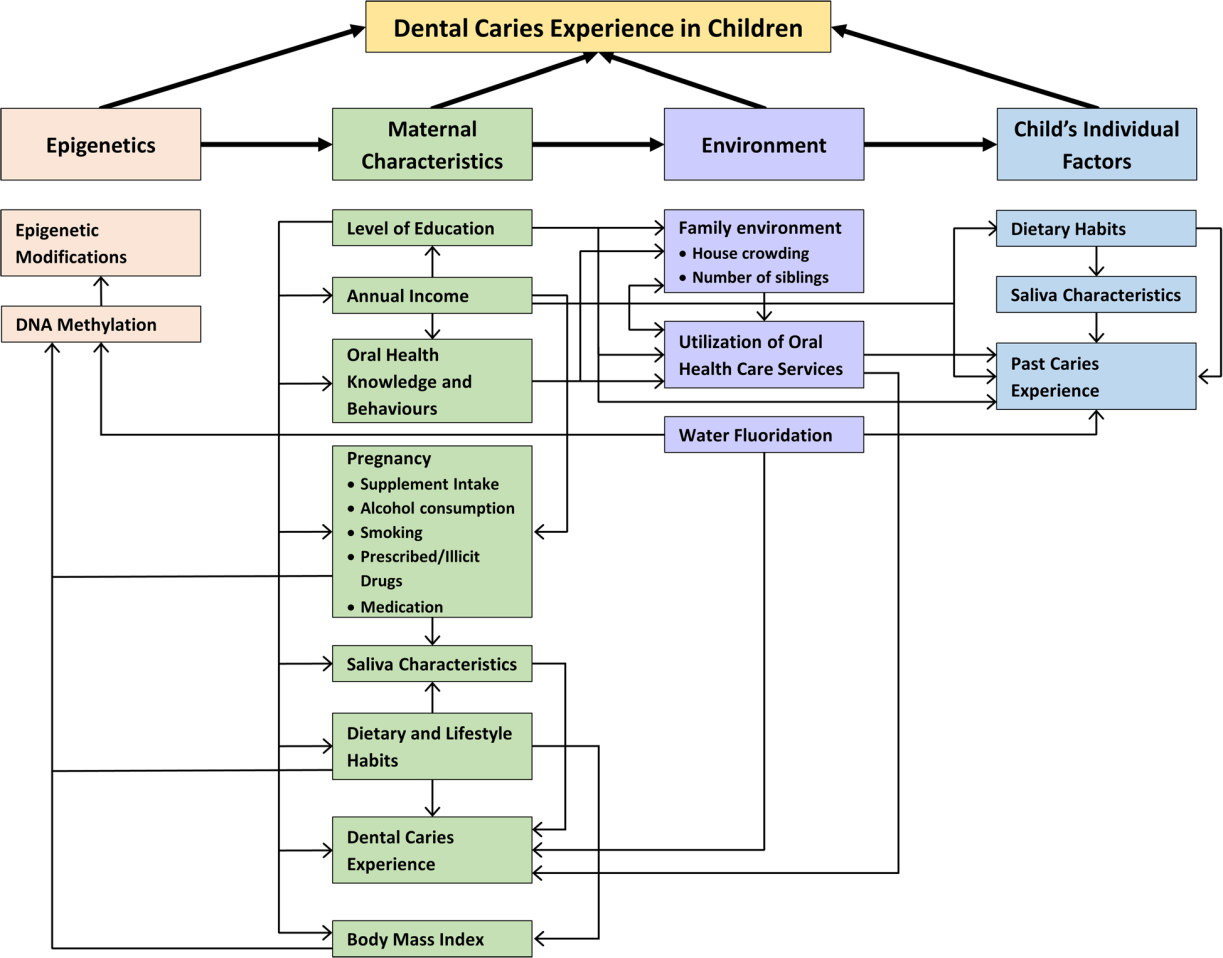
Conceptual framework for the possible interaction of risk factors/indicators under investigation

Oral health is determined by many factors: socio-economic [9], environmental [10], political [11], availability of oral health care facilities [12] and their level of utilization [13]. Furthermore an individual’s health related behaviours [14, 15], as well as oral health knowledge [16], attitudes [17] and practices [16], biological factors [18] including the intrauterine environment [19] and genes [20] have an impact on his/her oral health. Additionally, maternal factors like mother’s level of caries [21, 22], her oral health knowledge [16, 19, 22–25], salivary loads of cariogenic bacteria [26–28], salivary characteristics including pH, flow rate and buffering capacity [29, 30], as well as maternal genetics [20] are significant risk indicators to be considered for childhood dental caries. Furthermore, an holistic approach in identifying risk factors risk indicators for dental caries is required as oral health is but a part of general health [31]. Investigating newly emerging risk factors and risk indicators for dental caries is crucial and these can be discovered by studying the natural history of disease and by using modern molecular-based approaches [32]. A conceptual model of how we envision that these factors might interact is presented in Fig. 1.

The importance of inherited factors in susceptibility to dental caries has been recognized for decades [33, 34]. Heritability is the proportion of phenotype variation explained by genetic factors. These have been estimated to account for 30–60 % (*p* ≤ 0.01) of the variation in caries scores for the permanent dentition and 54–70 % (*p* ≤ 0.01) for deciduous caries scores [35]. According to Shaffer et al. there is direct evidence for a genetic component in the aetio-pathogenesis of dental caries [36] but very little is known about the specific causal mechanisms [37]. There are many genes related to the composition and structure of dental enamel, inherited alterations in sugar metabolism and genetic regulation of salivary gland function. Unsurprisingly, no single host gene that directly regulates dental caries initiation or progression has been identified [38, 39]. Epigenetics however, may provide the missing link to these unanswered questions [40]. Epigenetics investigates the molecular mechanisms that link genetic and environmental cofactors to disease outcomes [41]. There are many complex mechanisms underlying epigenetic alterations such as DNA methylation, histone modification and gene regulation by non-coding RNA, of which DNA methylation is the most common [42]. Such changes may be heritable or could be environmentally modulated. DNA methylation seems to be the primary mechanism to suppress retro-transposable elements which are responsible for creating genetic variation and, on occasion, disease-causing mutations within the human genome. Because these elements remain partially methylated in sperm, there may be a prolonged period of transposition lasting a few generations before gene expression [43]. Possible epigenetic contributions are currently being investigated in many diseases, but such studies are relatively novel in the dental field [42] and are needed to fully understand DNA-based factors important for development of oral diseases [31].

## Methods

### Study population

The research described in this paper is the oral health arm of the main “Environments for Healthy Living – the Griffith Birth Cohort study” (EHFL) which, beginning in 2006, has recruited some 3000 families from South East Queensland. EFHL is a prospective, multi-year longitudinal birth cohort study, collecting information during pregnancy through infancy and adulthood [43]. The EFHL study population includes all births from three geographically defined adjoining Health Districts in Queensland (Logan, Beaudesert and the Gold Coast) and Northern Rivers/Tweed in NSW from 2006 to 2011. These four areas cover 30 % of Queensland’s population. Women waiting for antenatal clinic appointments from the three public maternity hospitals (Logan, Gold Coast and Tweed) in the participating districts were contacted by research-trained midwives, provided with a detailed explanation of the study aims, and invited to participate [44]. Written informed consent was obtained from participants to access their information from hospital data bases, to complete a maternal baseline survey and for individual follow-up [44]. The initiators of the EFHL study expected to incorporate future sub-studies including research on dental and oral health [45]. The population for the oral health sub-study, which began in 2012 is, a subset of 6–7 year old EFHL children and their mothers who accept our invitation to participate.

Ethics approval to initiate the oral health sub-study was obtained from the Griffith University Human Research Ethics Committee on 26.12.2012, based on a full National Ethics Approval Form process, with NW Johnson as responsible Principal Investigator. A Variation to archive and analyse DNA was approved on 17.07.2014 (GU Ref No: OTH/25/13/HREC). The ethics approvals and informed consents obtained cover the clinical examination of mothers and their child/children and parental consent for full participation for their child in all aspects of the study.

### Sample recruitment and examination

**Fig. 2:**
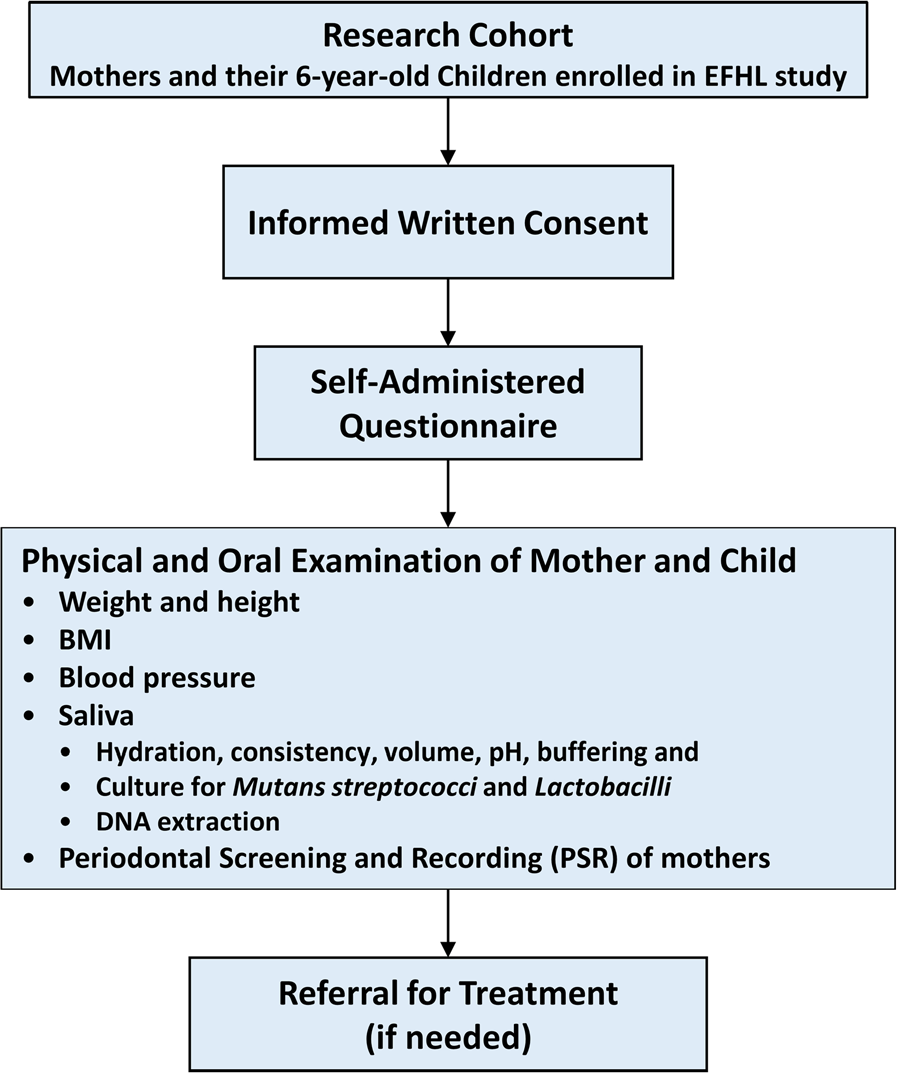
Flow chart showing the process of data collection

In 2012, mothers of the first cohort of children, who were 6 years old at the time, were sent letters alerting them to the oral health study, followed by a telephone call to assess their level of interest. Since then we went on to recruit participants from 2007 to 2009 cohorts and the recruitments are still taking place. Participants who find it difficult to travel far, who have serious illness and who are not willing to undergo a detailed oral examination are excluded from the study. Those who agree to attend the dental clinic at Griffith Dental School are given appointments for a free dental examination of the mother and child by two qualified dental surgeons, which includes offer of free treatment for the child, if required. Both mother and child are asked not to brush their teeth 2–3 h prior to examination, not to consume sweet food or beverages, smoke or use a mouth wash as per oral examination criteria. Mothers who are pregnant or who are wearing cardiac pace makers are excluded from weighing on the bio impedance scale. Detailed medical histories are taken when subjects arrive at chair side (Fig. 2). A careful examination of the head and neck, visually and by palpation, is performed. Each participant is then examined for salivary physiology, for the health of the oral soft tissues, for dental status and for experience of dental caries and periodontal disease (Fig. 2). Data are entered into the Titanium clinical management software (Spark Dental Technology, New Zealand) of the Griffith University Dental Clinic, incorporating pages specifically designed for the recording of research observations. Participants who are in need of treatment are referred for treatment at Griffith University dental clinics, that for children being free, the costs being covered by the Queensland Government.

### Sample size

Sample size required for a descriptive multivariate regression model depends on the probabilities of Type I and II errors, the effect size and its variation, the number of independent variables and correlation coefficients [46] and other factors. Methods of calculation tend to be complex; a simple equation for the comparison of two means has only just recently been published [47]. Power analysis of smaller multivariate models can be performed but it requires synthetic data based on an assumed model, model parameters and expected covariate distributions. A simple sample size table is available on https://www.statstodo.com/SSizMReg_Tab.php which requires an a priori knowledge of multiple correlation coefficient and the number of independent variables in the model. Another rule-of-thumb [48] applicable for this type of study considers the number of ‘candidate’ independent variables, interaction and other terms used during analysis, and requires the sample size to be 10–20 times this number. Hence, the minimum sample size requirement (*n* = 147 dyads) was initially guided by the one-sample correlation test to determine whether a correlation coefficient differs from zero, set at a low correlation level of *r* = 0.4 (two-tailed alpha = 0.05, power = 0.80), calculated using the “power one correlation” command of Stata v 13.1 data analysis and statistical software (STATACorp, United States) [49]. In this study the sample size is limited by pragmatic factors (budget, time and available sample population).

### Primary outcome variable

Each child’s dental caries experience is assessed using the International Caries Detection and Assessment System (ICDAS) [50]. The dentition is examined in detail, and every surface of every tooth scored after drying. Lesions of dental caries on both smooth surfaces and in pits and fissures, are scored from a range of 0 to 6 depending on severity. These scores cover a range from non-cavitated “white spot” and “brown spot” lesions to overt cavities. A special feature of ICDAS is the additional information it gives on the presence of, and type of, any restorations on every surface which would be counted as past caries experience. Overall caries experience per individual is calculated as a percentage of surfaces affected out of all surfaces examined, and this can be set at any level of caries severity.

### Main explanatory variables

Mother and child related variables are collected using questionnaires, clinical examinations and laboratory testings.

### Oral health knowledge and practices

After informed consent, a detailed questionnaire with 43 questions in total (developed for the study), related to oral health is administered to mothers before the clinical examinations, to collect data on knowledge, attitudes and practices towards oral health. Mothers are questioned on their knowledge of oral diseases, the causes of dental caries in children and adults and available oral health care services. With close-ended questions their dietary practices, oral hygiene methods and frequency, use of oral health care services and infant feeding practices are assessed. A Likert scale measures the attitudes towards oral health, and the importance mothers place on child’s oral health and deciduous teeth.

### Anthropometric measurements

Height, waist circumference and stride length of mother and child are measured. Participants are weighed with an electronic Bio-Impedance balance, which gives measurements of body fat, body water composition and body mass index (BMI) for adults and only weight for children. Mothers who are pregnant or who have a cardiac pacemaker implanted are excluded from weighing on the Bio-Impedance balance.

### Saliva characteristics

We evaluate oral hydration and salivary viscosity (sticky/frothy, frothy/bubbly or watery) by visual observation (Fig. 2). Stimulated saliva is collected over a period of 5 minutes by chewing on paraffin wax, and dribbling into a sterile cup. The expectorated volume is recorded. The pH and buffering capacity of stimulated saliva are measured using Saliva-check BUFFER kits (GC, United States of America) [51, 52]. Further, Caries Risk Test (CRT) kits (Ivoclar Vivadent, Australia) are used to assess saliva buffering and salivary counts of *Mutans streptococci* and *Lactobacilli* [52] (Fig. 2).

### Periodontal status

Mothers are examined for periodontal status using the Periodontal Screening and Recording (PSR) index, which measures the depth of periodontal pockets from the free gingival margin to the bottom of the gingival sulcus [53, 54]. Both the upper and lower teeth are divided into sextants. Each and every tooth in the oral cavity is examined and the highest score in each sextant is recorded. The scores vary from 0, denoting healthy periodontium, to 4, representing severe periodontitis [55].

### Demographic and environmental data

Demographic, social and environmental data were collected using detailed questionnaires during recruitment and follow-up phases of the EFHL study from 2006 to 2011. All selected factors are hypothesized predictors of the primary outcome. The present study will use the data on each family’s socioeconomic and demographic information of the mother: maternal age, mother’s highest level of education, employment status and total annual family income. The child’s intrauterine environment will be assessed by maternal prenatal nutrition, mother's smoking habits prescribed and illicit drug use and alcohol. Data on breast feeding and weaning practices, types of food and beverages consumed, infant supplementary food and bottle feeding practices, will be used as covariates to include in the risk factor model. Oral and other health care received by the child will be assessed from data on dental and medical check-ups (GP visits), emergency department attendances and availability of a medical insurance (health care card).

### Genetic and epigenetic markers

To identify genetic and epigenetic markers associated with dental caries we will target host genomic DNA obtained from saliva samples and use next-generation sequencing technology to measure DNA-based variants on a genome-wide scale.

Specifically, stimulated saliva will be collected in 2 ml tubes and stored at -80oC until DNA is extracted with the High Pure PCR Template Preparation Kit (Roche Diagnostics, Australia) using a method which was extensively modified in order to significantly increase the DNA yield to a concentration needed for next-generation sequencing (unpublished protocols). The extracted DNA will be sent to the Centre for Clinical Genomics at University of Queensland to perform sequencing of bisulfite treated DNA for methylation analysis. This approach will allow us to gain information on both DNA sequence variants and CpG methylation at ~450,000 CpG sites spanning the human genome. These assays will be performed using the SeqCap Epi 4 M CpGIANT Enrichment Kit (Roche NimbleGen, Australia) and the HiSeq2500 Sequencer (Illumina, United States) [56]. A manuscript by Allum et al. provides a detailed description of the assay protocols [57].

To date we have conducted a pilot study with 12 mother child dyads; 6 with high caries experience (average caries experience, 27.1 % of surfaces affected in mothers and 12.4 % in children) and 6 with low caries experience (average caries experience, 11.2 % in mothers, 2.5 % in children) was initiated using data collected within the oral health sub study of EFHL. As a result of this pilot study we have been able to establish the DNA extraction, purification and sequencing protocols (in our hands) and have also established the necessary bioinformatics and statistical analysis methods for identifying target genes for dental caries.

### Analysis

Observations will be stored as de-identified data, using a unique identifier for each participant. The dataset for the analysis will be prepared in SPSS in a wide data format (one row per participant). Data cleaning will be performed. Outliers will be investigated and corrected or replaced with missing values if necessary. The pattern of missing values will be investigated, and variables will not be used for further analysis if >30 % of values are found missing; missing data will not be imputed.

The dependent variable (dental caries experience of children) is expected to be a ratio type continuous variable, right-skewed with a mode of zero and which could be modelled using a generalised linear model (GLM) with log-link and gamma family. The number of independent variables will be reduced where possible (e.g. oral health knowledge and practices, periodontal status, etc.) by using aggregate values. Independent variables without a known or hypothesised association with the dependent variable will not be used in regression analyses. The independent variables (covariates) will be prepared as follows. Dichotomous variables: coded as 0/1; the zero category with the higher frequency; presented as number and proportion (%); ignored for GLM if rare (frequency < 20). Categorical variables: coded as 0/1/2/etc.; sorted by frequency in a descending order; presented as number and proportion; for GLM the smaller categories will be collapsed to form categories with frequency > 20; dummy coded for GLM. Ordinal variables: coded as 0/1/2/etc.; sorted in a meaningful way; presented as number and proportion; for GLM neighbouring categories will be collapsed to form categories with *n* > 20. Continuous variables: re-scaled where necessary to ensure a change of a value of 1 can be interpreted in a meaningful way; centred over mean where necessary (eg. for age); presented as mean and standard deviation (symmetrical distributions) or median and 25th/75th percentiles.

Descriptive statistics of participant characteristics (of values before re-categorisation or transformation) will be presented. Unadjusted associations between dependent and covariates will be explored with bivariate GLMs. The covariates of interest for a multivariate model will be shortlisted at *p* < 0.2. Correlations between the shortlisted covariates will be investigated (using Pearson or Spearman correlation and scatter plots), and covariates will not be used together in a multivariate model if a statistically significant correlation is found at |r| > 0.4. Scatter plots will be drawn to investigate the relationships between the dependent variable and the covariates. Interaction terms will be created where necessary. The multivariate GLM will be built by manually adding covariates one-by-one (forward method; sorted by descending univariate p-values), whilst observing changes to the coefficients and errors for variables already in the model. Covariates will be dropped from the multivariate model at *p* > 0.05. Collinearity between covariates will be checked with the variance inflation factor. Model building will be repeated using a backward method to check the final model. The number of covariates in the model will be limited to avoid overfitting, as discussed in the sample size section. Assumptions of GLM will be checked and the model adjusted if necessary. Coefficients will be presented with the 95 % confidence intervals, and evaluated considering clinical significance. Statistical significance will be declared at *p* < 0.05; the effect of multiple comparisons on overall error rates will be considered during discussion of the results.

For epigenetic variation analysis, site specific methylation levels will be defined as a percentage methylation, with values ranging between 0 between 1. Each identified differentially methylated site will be attributed a percentage methylation that will be included in the regression modelling approach. Pyrosequencing on selected differentially methylated sites will be done to validate the Seq Cap Epi data.

## Discussion

This cross-sectional study to assess maternal, environmental and individual risk factors for childhood dental caries will examine a wide range of determinants. It will evaluate inter-relationship among main effects and their relative effects on dental caries of children. Furthermore, this research will pioneer research in epigenetic variation for dental caries in children. The study will also potentially provide evidence on the interrelationship of epigenetic variations with other social and environmental predictors for dental caries in the participating child population. The evidence generated by the current study will enable a more effective risk factor modification approach to overcome the problem of this chronic disease that commences in childhood and our data can be extrapolated to similar populations worldwide. From thereon, we expect that appropriate interventions will be recognized and implemented to tackle dental caries in children and improve oral health and quality of life of the affected. However, due to time and logistic constraints, obtaining a larger sample size is one limitation of the study.

## Abbreviations

BMI: Body mass index
ICDAS: International Caries Detection and Assessment System
DNA: Deoxyribose Nucleic Acid
dmft: decayed missing and filled teeth index for deciduous teeth
SEQ: South East Queensland
EFHL: Environments For Healthy Living
NEAF: National Ethics Approval Form
CRT: Caries Risk Test
